# Pharmacological inhibition of syntenin PDZ2 domain impairs breast cancer cell activities and exosome loading with syndecan and EpCAM cargo

**DOI:** 10.1002/jev2.12039

**Published:** 2020-12-15

**Authors:** R. Leblanc, R. Kashyap, K. Barral, A.L. Egea‐Jimenez, D. Kovalskyy, M. Feracci, M. Garcia, C. Derviaux, S. Betzi, R. Ghossoub, M. Platonov, P. Roche, X. Morelli, L. Hoffer, Pascale Zimmermann

**Affiliations:** ^1^ Equipe labellisée Ligue 2018 Centre de Recherche en Cancérologie de Marseille (CRCM) Aix‐Marseille Université, Inserm, CNRS, Institut Paoli‐Calmettes Marseille France; ^2^ Centre de Recherche en Cancérologie de Marseille Integrative Structural & Chemical Biology Aix‐Marseille Université, Inserm, CNRS, Institut Paoli Calmettes Marseille France; ^3^ Enamine Ltd. Kyiv Ukraine; ^4^ Taras Shevchenko National University of Kyiv Kyiv Ukraine; ^5^ Institute of Molecular Biology and Genetics National Academy of Sciences of Ukraine Kyiv Ukraine; ^6^ Department of Human Genetics K. U. Leuven Leuven Belgium

**Keywords:** drug design, exosomes, inhibitors, PDZ, syndecan, Syntenin

## Abstract

Exosomes support cell‐to‐cell communication in physiology and disease, including cancer. We currently lack tools, such as small chemicals, capable of modifying exosome composition and activity in a specific manner. Building on our previous understanding of how syntenin, and its PDZ partner syndecan (SDC), impact on exosome composition we optimized a small chemical compound targeting the PDZ2 domain of syntenin. In vitro , in tests on MCF‐7 breast carcinoma cells, this compound is non‐toxic and impairs cell proliferation, migration and primary sphere formation. It does not affect the size or the number of secreted particles, yet it decreases the amounts of exosomal syntenin, ALIX and SDC4 while leaving other exosomal markers unaffected. Interestingly, it also blocks the sorting of EpCAM, a *bona fide* target used for carcinoma exosome immunocapture. Our study highlights the first characterization of a small pharmacological inhibitor of the syntenin‐exosomal pathway, of potential interest for exosome research and oncology.

## 1 INTRODUCTION

It is now clear that cells communicate via the release of membrane‐limited vesicles, collectively called extracellular vesicles (EVs) (Kalluri & Lebleu, 2020; Mathieu, Martin‐Jaular; Lavieu, & Théry, 2019; Stahl & Raposo, 2019). EVs represent complex biological messengers, containing lipids, proteins, and nucleic acids, including miRNAs. EVs appear to be important in various physio‐pathological situations, in particular during cancer progression, with a role in metastatic niche formation and tumour immune escape (Greening, Gopal, Xu, Simpson, & Chen, 2015; Wortzel, Dror, Kenific, & Lyden, 2019). Their use for biomarker discovery, and as vehicles or targets of therapies is currently the focus of intense research. Pending on their origin and mechanism of formation, specific subsets of these vesicles are generally referred to as microvesicles, exosomes, or apoptotic bodies among others. Exosomes are ‘exocytic’ vesicles, approximately 40–100 nm in size, released from most cell types. Their biogenesis starts by the inward budding of the limiting membrane of endosomes, generating multivesicular endosomes. Exosomes, including exosomes derived from both the tumour and tumour‐associated host cells, help composing the niches that support tumour development, occupying a central place in tumour biology. ‘Cancer exosomes’ are thus gaining considerable attention, in basic and applied clinical research, for their role in the modulation of the tumour microenvironment and their contribution to tumour‐host crosstalk.

Our laboratory has identified a molecular path, supported by the syndecan (SDC) heparan sulfate proteoglycans and their adaptor protein syntenin, which regulates the biogenesis of a specific class of exosomes (Friand, David, & Zimmermann, 2015). Syntenin is a small intracellular scaffold protein that contains two PSD‐95, Discs‐large, ZO‐1 (PDZ) domains that bind SDC, an N‐terminal domain that binds ALIX (via three LYPxL motifs), and a short C‐terminal domain regulating the conformation of the protein and accessibility of its domains (Baietti et al., 2012; Grootjans et al., 1997; Saito, Nishioka, Iwatsuki, Kumagai, & Sasaki, 1975). The PDZ domains of syntenin bind the C‐termini of the SDCs (Grootjans et al., 1997), but bind also to PIP2 (Zimmermann et al., 2002) and several other membrane proteins, including CD63 (Latysheva et al., 2006). The binding of syntenin to PIP2 regulates the recycling of endocytosed SDCs from late endosomes back to the cell surface (Zimmermann et al., 2005). In contrast, SDC‐syntenin‐ALIX binding regulates the formation of endosomal intraluminal vesicles and the secretion of CD63^+^ exosomes that contain syntenin, C‐terminal fragments of the SDCs (SDC‐CTFs) and potential SDC‐cargo such as ligand‐engaged receptors (Baietti et al., 2012). SDCs have been linked to a plethora of cellular processes, including cell–cell and cell–matrix adhesion, signal transduction, as well as vesicular trafficking (Christianson & Belting, 2014; Couchman, 2010; Lambaerts, Wilcox‐Adelman, & Zimmermann, 2009). The expression level of syntenin are extremely high during fetal development compared to adult tissues (Zimmermann et al., 2001). Noteworthy, gain of syntenin expression in tumour cells has been associated with the invasion and the metastatic potential of various solid cancers (Kegelman et al., 2015).

The precise mechanisms involved remain to be further clarified, but the correlation implies that syntenin‐exosomes might be implicated in the processes of malignant cell invasion and the establishment of niches that promote tumour metastasis. Along that line, we recently revealed that SRC oncogene functions in cell‐to‐cell communication by controlling the biogenesis of syntenin‐exosomes, identifying these as a specific class of pro‐migratory exosomes stimulating recipient HUVEC cells. SRC kinase, phosphorylating both SDCs and syntenin, supports the endosomal budding and exosomal secretion of syntenin and associated cargo, including syndecans and CD63, both binding directly to syntenin, and, in turn, components potentially bound to SDC and tetraspanins, such as integrins and EGFR (Imjeti et al., 2017). Similarly, the syndecan‐syntenin‐ALIX pathway is also known to impact on the intracellular trafficking and exosomal confinement of FGF signals (Baietti et al., 2012). Exosomal CD81, CD9 and flotillin, in contrast, do no depend on SDC‐syntenin (Ghossoub et al., 2014; Imjeti et al., 2017; Roucourt, Meeussen, Bao, Zimmermann, & David, 2015), underscoring the heterogeneity of exosome compositions and of mechanisms of exosome formation. Together, all the data therefore suggest that exosome biogenesis regulated by the syntenin‐syndecan pathway might significantly contribute to tumour‐host interactions.

Targeting protein–protein interactions (PPI) has emerged as a potential approach for the treatment of solid tumours or hematologic malignancies (Cierpicki & Grembecka, 2015; Makley & Gestwicki, 2013; Morelli, Bourgeas, & Roche, 2011). Promising results have been obtained with PPI inhibitors of p53 and BclxL or more recently bromodomain inhibitors (Faivre et al., 2020; Filippakopoulos et al., 2010; Jung, Gelato, Fernández‐Montalván, Siegel, & Haendler, 2015; Milhas et al., 2016; Ulucan, Eyrisch, & Helms, 2012). PDZ interactions are intracellular PPIs that occur between so‐called PDZ domains and short peptide sequences (PDZ binding motifs). Since syntenin activity strictly depends on the functionality of its PDZ domains (Baietti et al., 2012; Grootjans et al., 1997; Grootjans, Reekmans, Ceulemans, & David, 2000; Koo et al., 2002; Lambaerts et al., 2012; Luyten et al., 2008; Meerschaert et al., 2007; Zimmermann et al., 2002; Zimmermann et al., 2005; Zimmermann et al., 2001), we aimed to develop syntenin PDZ inhibitors and investigate their potential utility to control cellular phenotypes as well as exosomal‐releases. By screening a PDZ‐focused fragment library and pursuing a hit‐to‐lead optimization strategy, we identified a small molecule that selectively affects syntenin‐exosomal releases. We also provide evidence that this inhibitor markedly affects the loading of exosomes with EpCAM, a transmembrane glycoprotein involved in cell migration, adhesion, proliferation and differentiation, which has been associated with the processes of tumorigenesis and metastasis of several carcinomas (Keller, Werner, & Pantel, 2019).

## 2 MATERIALS AND METHODS

### 2.1 Compound library and in silico selection

As a source of screening compounds, we used the PDZ targeted library (1185 compounds at the time) developed at Enamine to cover all PDZ domains with published structures (https://enamine.net/hit-finding/focused-libraries/ppi-library). For this study, compounds from the PDZ targeted library were selected with two approaches. First, a random subset of 118 compounds was selected which represented 10% of the library. The second subset (an extra 21 compounds) resulted from docking of the PDZ library to crystal structures of syntenin PDZ2, yielding compounds selected specifically against the syntenin PDZ2 domain. For that, two crystal structures of the PDZ2 domain (PDB 1W9O, 1W9E) with bound peptides were selected for the docking stage. The 1W9E structure includes a small rearrangement of the site (essentially Phe213) allowing the binding of a larger C‐terminal hydrophobic residue (Val in 1W9O and Phe in 1W9E). The Schrödinger suite [http://www.schrodinger.com] was used for the modelling stage. Binding sites were defined as 20 Å cube centred on the carboxylic carbon from C‐terminal residue of the bound peptide. Compounds from the PDZ‐targeted library were converted into 3D format using LigPrep routine. The Glide docking tool was employed for the virtual screening of the library to both binding sites. Standard precision level of accuracy was used and a maximum of five poses per ligand were allowed during the virtual screening stage. The final selection of compounds was based on three criteria: score from Glide scoring function, visual inspection of poses and ability of a compound to partially mimic binding mode of the C‐terminal residue from corresponding peptide ligand. At least one hydrogen bond with backbone atoms of Val209 or Phe211 was required. A total of 25 compounds were finally selected, after the visual analysis stage, to define the second set of compounds to be experimentally evaluated. In total, 139 non‐redundant compounds were selected in silico for further analysis.

### 2.2 Molecular biology

Human syntenin1 cDNA was cloned into the pGEX‐5X vector (residues 1–298 or syntenin full‐length (FL) for HTRF assays) (Amersham Pharmacia Biotech AB) or into the pETM11 vector (residues 113–273 of syntenin used for crystallization) (EMBL Heidelberg) to produce N‐Terminal GST or 6xHis tagged fusion proteins respectively. Human GRASP55 tandem PDZ and human Erbin PDZ domain were cloned into the Gateway pDEST15 prokaryotic expression vectors (Invitrogen) intended to produce the corresponding N‐terminal GST‐tagged fusion protein, as previously described (Milhas et al., 2016). For BIAcore experiments, the constructs pAviTag_N‐His_syntenin tandem + Cterm (residues 107–298), pAviTag_N‐His_syntenin tandem (residues 107–275), pAviTag_N‐His_GRASP tandem (residues 1–209) and pAviTag_N‐His_GST tagged with 6xHis at the N‐terminus, followed by the biotinylation site (AviTag peptide, GLNDIFEAQKIEWHE), were generated by recombination between the pAviTag_N‐His vector (Lucigen) and the PCR amplification product from appropriate cDNA libraries, using primers including 18 nucleotides of overlap with the ends of the vector (sense 5′‐CCG AGC ACT CCT CCT ACC and antisense 5′‐GTG GCG GCC GCT CTA TTA). All open reading frames were verified by DNA sequencing.

## 3 PROTEIN PRODUCTION AND PURIFICATION

### 3.1 6xHis‐syntenin (aa 113–273) for crystallization studies

Competent E.coli (ER2566 strain) cells were transformed with the pETM‐11 vector (EMBL Heidelberg) allowing expression of N‐terminally His‐tagged proteins. Bacteria were plated on LB agar plates with 50 µg/ml Kanamycin. The culture and induction were performed in TYB medium (5‐6 h induction at 30°C after addition of 0.5 mM IPTG). The pellet was re‐suspended in 30 ml of lysis buffer (25 mM Hepes pH 7.4, 150 mM NaCl, 1 µg/ml DNAse I Recombinant RNase free (Qiagen), 0.013 g Lysozyme (Sigma) and 1 tablet of protease inhibitor EDTA free (Roche)) and kept on ice for 30 min. After sonication, the suspension was transferred to JA 20 tubes and centrifuged at 12,000 × *g* at 4°C for 45 min. The supernatant was applied to a HistrapTMHP columns 17‐5247‐01 (GE Healthcare) equilibrated with 20 ml Buffer A (25 mM Hepes pH 7.4, 150 mM NaCl and 20 mM Imidazole). The purification was performed on the Akta Explorer (Amersham Pharm Biotech). His‐tagged proteins were eluted with 30 ml of an imidazole gradient (20–500 mM, Buffer B, 25 mM Hepes pH 7.4, 150 mM NaCl and 500 mM Imidazole). 6xHis‐tagged proteins were fully recovered within the first 10 ml of elution. The His‐tag was removed using TEV protease at 4°C for 16 h for crystallization trials. Purification was finalised using size exclusion gel chromatography with a HiLoad 16/600 Superdex 75pg (GE Healthcare) column in buffer containing 10 mM Hepes pH 7.4, 150 mM NaCl and 1 mM DTT.

### 3.2 GST‐GRASP55 and GST‐Erbin PDZ domains for HTRF experiments

The production of N‐terminal GST‐tagged GRASP55 fusion proteins and N‐terminal GST‐tagged Erbin were accomplished by an induction 18 h at 25°C with 0.4 mM IPTG in E. coli BL21 (DE3) bacteria cells transformed with the purified pDEST15‐GRASP55 or pDEST15‐Erbin expression plasmids (Invitrogen). Fusion proteins were recovered from the cell lysates by conventional affinity chromatography on Glutathione resin using an Akta Explorer system (Amersham Pharm Biotech).

### 3.3 GST‐syntenin full length for HTRF experiments

Competent E. coli (ER2566 strain) cells were transformed with the human syntenin1 pGEX‐5X expression vector (GE Healthcare). Expression of N‐terminally GST‐tagged syntenin‐1 full length was induced overnight at 30°C and by the addition of 0.4 mM IPTG (Fisher Scientific). Protein was purified using GSTrap4B columns 28‐4017‐45 (GE Healthcare).

### 3.4 Biotinylated 6xHis tag fusion proteins for BIAcore experiments

Competent Biotin XCell F’ cells were transformed with pAviTag_N‐His_syntenin tandem + Cterm, pAviTag_N‐His_syntenin tandem, pAviTag_N‐His_GRASP tandem or pAviTag_N‐His_GST expression vectors. Expression of the protein was induced overnight at 25°C by addition of rhamnose, arabinose and biotin at final concentrations of 0.2%, 0.01% and 50 µM, respectively. Proteins were purified using HistrapTMHP columns 17‐5247‐01 (GE Healthcare).

### 3.5 HTRF screen

HTRF assays were performed in white 96Well Small VolumeHiBase Polystyrene Microplates (Greiner) with a total working volume of 100 µl, as described previously (Milhas et al., 2016). Briefly, compounds were firstly dispensed into the wells at 400 µM for the primary screen or with serial DMSO dilutions for IC_50_ measurement assays. Primary screening assays have been performed in monoplicate while IC_50_ measurements were performed in triplicates. All HTRF reagents were purchased from CisBio Bioassays and reconstituted according to the supplier protocols. The plates were spun for 3 min at 500 × *g* and then incubated at 4°C for 16 h. The HTRF signals were recorded on a POLAR star Omega plate reader (BMG Labtech) with an excitation filter at 337 nm and fluorescence wavelength measurement at 620 and 665 nm, an integration delay of 60 µs and an integration time of 400 µs. Results were analysed with a two‐wavelength signal ratio: [intensity (665 nm)/intensity (620 nm)] × 10^4^. Percentage of inhibition was calculated using the following equation: % inhibition = [(compound signal)–(min signal)]/[(max signal)–(min signal)] × 100, where ‘max signal’ is the signal ratio with the compound vehicle alone (DMSO) and ‘min signal’ the signal ratio without protein B. For IC_50_ measurements, values were normalized and fitted with Prism (GraphPad software) using the following equation: Y = 100/(1+((X/ IC_50_)ˆHill slope)).

### 3.6 Surface plasmon resonance experiments

All SPR experiments were conducted with a BIAcore T200 instrument (GE Healthcare), at 25°C. A total of 8000 resonance units (RU) of biotinylated ligands (N‐terminal 6xHis tagged biotinylated syntenin tandem, syntenin tandem with C terminus, GRASP55 tandem or GST as control) were immobilized on a streptavidin‐sensor chip (GE Healthcare). Analytes (C58 and SyntOFF compounds) were perfused at 30 µl per min in running buffer (10 mM HEPES pH 7.4, 150 mM NaCl, 0.005% Tween20) at different concentrations. The reference channel was N‐terminal 6xHis tagged biotinylated GST protein. The injection time was 240 s, long enough for the sensorgrams to reach equilibrium, and the dissociation time was 240 s. To carry out these SPR experiments, we employed the method known as ‘single‐cycle kinetics’ described in 2006 by Karlsson and co‐workers (Karlsson, Katsamba, Nordin, Pol, & Myszka, 2006). It consists in injecting the analyte at increasing concentrations, without regeneration steps between each sample injection to avoid that folded‐proteins can be damaged after regeneration steps. Equilibrium dissociation constants (K_D_) were calculated by fitting the data to a simple Langmuir binding isotherm using GraphPad Prism. For determination of the apparent K_D_, signals obtained at equilibrium were plotted as a function of compound concentration. Cognate peptides for syntenin (19mer SDC4 peptide, sequence YDLGKKPIYKKAPTNEFYA) and GRASP55 (19mer JAM‐C peptide, sequence NYIRTSEEGDFRHKSSFVI) were used to confirm the functionality of the immobilized proteins.

### 3.7 Chemistry

All commercial reagents were purchased from Sigma‐Aldrich company. Furthermore, all dry solvents were obtained via Sigma‐Aldrich with Sure/Seal system and regular solvents were obtained via Sigma‐Aldrich at technical grade. Analytical thin layer chromatography (TLC) of the reactions was performed on silica gel 60F 254 aluminium plates (Merck) of 0.2 mm thickness with appropriate solvents. The spots were examined with UV light (λ = 254 nm). Preparative flash column chromatographie's were performed using silica gel (Merck) G60 230–240 under compressed air. The ^1^H NMR and ^13^C NMR spectra were determined with a BRUCKER AMX 250 MHz or BRUKER Avance III nanobay 400 MHz. The chemical shifts are reported in ppm and coupling constants (*J*) are reported in hertz. Reaction monitoring and purity of compounds were recorded by analytical Agilent Infinity high performance liquid chromatography (Column Zorbax SB‐C18 1.8 µM (2.1 × 50 mm); Mobile phase (A: 0.1% FA H_2_O, B: 0.1% FA CH_3_CN, Time/%B: 0/10, 4/90, 7/90, 9/10, 10/10); Flow rate 0.3 ml/min with DAD at 254 nM. All tested compounds yielded data consistent with a purity of ≥95%. Low‐resolution mass spectra were obtained with Agilent SQ G6120B mass spectrometer in positive and/or negative electrospray modes. Resolution Mass Spectra (HRMS) were obtained on a SYNAPT G2‐S WATERS mass spectrometer.

### 3.8 Synthesis of 3‐(4‐chlorophenylthio)propanoic acid 2

To a stirred solution of 4‐chlorothiophenol 1 (1 eq, 5.0 mmol) and sodium hydroxide (2.4 eq, 12.0 mmol) in water, aqueous solution of 3‐chloropropionic acid was added dropwise (1.7 eq, 8.5 mmol). The mixture was refluxed during 3 h. After completion, 1N HCl solution was added dropwise at 4°C until pH 1, to afford a crude precipitate which was filtered and dried. The crude precipitate was purified by column chromatography eluting with dichloromethane‐methanol (100 to 98:2) to afford pure compound **2**. ^1^H NMR (CDCl_3_, 250 MHz) δ = 7.35–7.08 (m, 4H), 3.07 (t, *J* = 7.2 Hz, 2H), 2.59 (t, *J* = 7.2 Hz, 2H). ^13^C NMR (CDCl_3_, 63 MHz) δ = 175.58, 132.74, 132.16, 130.80, 128.18, 33.13, 28.39. LC/MS (ESI): 215.7 [M‐H]^–^; white powder. Yield = 75%.

### 3.9 Synthesis of (S)‐methyl 2‐(3‐(4‐chlorophenylthio)propanamido)‐3‐methylbutanoate cSyntOFF

To a stirred solution of propionic acid derivative **2** (1 eq, 1.0 mmol) and L‐valine methyl ester hydrochloride (1 eq, 1.0 mmol) in dry dichloromethane, N‐Ethyl‐N′‐(3‐dimethylaminopropyl)carbodiimide hydrochloride (2 eq, 2.0 mmol) and triethylamine (2 eq, 2 mmol) were added. The mixture was stirred under argon atmosphere during 16 h at room temperature. After completion, the resulting mixture was washed twice with 1N HCl solution, once with water, once with brine, dried over Na_2_SO_4_, and evaporated under vacuum. The crude residue was purified by column chromatography eluting with dichloromethane‐methanol (100 to 98:2) to afford pure compound **cSyntOFF**. ^1^H NMR (DMSO*‐d^6^*, 300 MHz) δ = 8.19 (bd, *J* = 8.1 Hz, 1H), 7.38 (d, *J *= 8.9 Hz, 2H), 7.35 (d, *J *= 9.0 Hz, 2H), 4.26–4.12 (m, 1H), 3.63 (s, 3H), 3.14 (t, *J* = 7.1 Hz, 2H), 2.53 (t, *J* = 7.1 Hz, 2H), 2.00 (m, 1H), 0.87 (d, *J* = 6.6 Hz, 3H), 0.86 (d, *J* = 6.7 Hz, 3H). ^13^C NMR (DMSO*‐d^6^*, 75 MHz) δ = 172.47, 170.86, 135.62, 130.89, 130.45, 129.41, 57.87, 52.06, 34.91, 30.39, 28.83, 19.40, 18.72; LC/MS (ESI): 328.63 [M‐H]^–^, 330.1 [M+H]^+^; white solid. Yield = 62%.

### 3.10 Synthesis of (S)‐2‐(3‐(4‐chlorophenylthio)propanamido)‐3‐methylbutanoic acid SyntOFF

To a stirred solution of ester intermediate derivative **3** (1eq, 0.5 mmol) in THF at 0°C, aqueous solution of LiOH (10 eq, 5.0 mmol) was added dropwise. The resulting mixture was stirred 1 h at room temperature. After completion, THF was removed under vacuum. Then, 1N HCl solution was added dropwise until pH 2–3 and the aqueous layer was extracted with ethyl acetate. The organic layer was washed once with water, once with brine, dried over Na_2_SO_4_, and evaporated in vacuo to afford crude residue **SyntOFF** which does not require further purification. ^1^H NMR (DMSO*‐d^6^*, 300 MHz) δ = 12.51 (bs, 2H), 8.04 (d, *J* = 8.5 Hz, 1H), 7.38 (d, *J* = 9.2 Hz, 2H), 7.34 (d, *J* = 9.3 Hz, 2H), 4.16 (dd, *J* = 8.4 Hz and 6.0 Hz, 1H), 3.14 (t, *J* = 7.1 Hz, 2H), 2.54 (t, *J* = 7.0 Hz, 2H), 2.03 (m, 1H), 0.87 (bd, *J* = 6.6 Hz, 6H); ^13^C NMR (DMSO*‐d^6^*, 75 MHz) δ = 173.41, 170.71, 135.69, 130.86, 130.42, 129.40, 57.62, 34.99, 30.34, 28.91, 19.58, 18.48. LC/MS (ESI): 314.6 [M‐H]^–^, 316.2 [M+H]^+^; HRMS (TOF, ESI+) calculated for C_14_H_19_NO_3_SCl [M+H]^+^ 316.0774, found 316.0776; white solid. Yield = 89%.

### 3.11 X‐ray crystallography

Crystallization trials were performed using the hanging drop vapour diffusion method at 4 and 12°C. The apo–Syntenin PDZ protein crystallized in 0.1 M Sodium Acetate pH 4.6/0.2 M Ammonium Acetate and 22% PEG 3350 at a protein concentration of 6 mg/ml. Syntenin PDZ crystals were soaked in mother liquors containing 5 mM of **SyntOFF** or 5 mM **C58** compounds (5% DMSO) for 4 h at 12°C. Crystals were flash‐frozen in mother liquor containing 10% glycerol. Data set of syntenin PDZ ‐ **SyntOFF** and Syntenin PDZ ‐ **C58** were collected at the ID30B ESRF and processed using X‐ray Detector Software (XDS).

### 3.12 Compound C58

Data set were collected at a resolution of 1.91 Å and cut at 2.0 Å. The space group was assigned to P1 with four proteins and two **C58** compounds per asymmetric unit. The phase was solved by molecular replacement using the structure of the PDB entry 1W9E as a template and the program PHASER. Models were rebuilt using COOT and refined with REFMAC5.

### 3.13 Compound SyntOFF

Data set were collected at a resolution of 2.01 Å and cut at 2.2 Å. The space group was assigned to P1 with four proteins and four **SyntOFF** compounds per asymmetric unit. The phase was solved by molecular replacement using the structure of the PDB entry 1W9E as a template and the program PHASER. Models were rebuilt using COOT and refined with REFMAC5. Both datasets showed translational Non‐Crystallographic Symmetry (TNCS) that was handled by PHASER during the molecular replacement step.

### 3.14 Cell culture and reagents

MCF‐7 cell lines were purchased from the American Type culture collection (Manassas, VA, USA). Cells were grown in DMEM‐F12 (Invitrogen) supplemented with 10% fetal calf serum (FCS) (Eurobio) at 37°C under 5% CO_2_. Syntenin CRISPR/Cas9 knockout (SyntKO) MCF‐7 cells were generated following the procedures as previously described (Imjeti et al., 2017). Briefly, SDCBP‐specific gRNA oligos sgRNASDCBP1 targeting exon1 (F: CACCgCTATCCCTCACGATGGAAGT; R: AAACACTTCCATCGTGAGGGATAGc) were cloned into the pX458 two‐in‐one CRISPR targeting vector (Addgene, Cambridge, MA, USA), individually transfected into MCF7 cells and sorted for GFP expression after 48 h. Single cell clones were grown and screened by western blotting for syntenin expression. One individual clone has been used in the in vitro experiments. The cDNA encoding mCherry‐syntenin was derived from the eGFP‐syntenin cDNA construct (Grootjans et al., 1997), by restriction‐ligation. The expression vector for Ce‐RAB5(Q79L) was received from W. Annaert (K.U. Leuven, Belgium). The CAY10594 inhibitor (used at 10 µM) was purchased from Santa Cruz (sc‐223874).

### 3.15 Cancer cell viability

The effects of the compounds **C58** and **SyntOFF** were tested both on MCF‐7 and MCF‐7 SyntKO cells. Briefly, the cells were treated with increasing concentrations of the compounds for 48 h and early/late apoptosis was measured by using the FITC Annexin V apoptosis detection kit with 7‐AAD (Biolegend, San Diego, CA, USA) according to the manufacturer. Annexin V positive but 7‐AAD negative (early apoptotic cells) and Annexin V positive and 7‐AAD positive (late stage apoptosis) was determined by using FACS LSRII flow cytometer (BD Biosciences) and data were analysed with Flowjo software (Tree Star).

### 3.16 Exosomes and total cell lysates

For comparative analyses, exosome‐enriched fractions were collected from equivalent amounts of culture medium, conditioned by equivalent amounts of cells. MCF‐7 cells were treated with indicated compounds or with DMSO 0.2% as control, in medium containing exosome‐depleted FCS (10%). After 16 h, cell media were collected and exosomes were isolated by three sequential centrifugation steps at 4 °C: 10 min at 500 × *g*, to remove cells; 30 min at 10,000 × *g*, to remove cell debris; and 1h30min at 100,000 × *g*, to pellet exosomes (exosome‐enriched fraction), followed by one wash with 1400 µl of PBS1X (100,000 × *g*, 1 h), to remove soluble serum and secreted proteins. Exosomal pellets were then re‐suspended in 100 µl of PBS1X. The lysates from corresponding cultures were cleared by centrifugation at 1500 rpm for 5 min and then resuspended in lysis buffer (TrisHCL pH 7.4 30 mM, NaCl 150 mM, 1% NP40 (IGEPAL), 1 µg/ml aprotinin, 1 µg/ml leupeptin). Although only little variations were observed from sample to sample, exosomal amounts loaded for the western blot were normalized according to the number of parent cells from where exosomes were secreted.

### 3.17 Western blots

The proteins were heat‐denaturated in Laemmli sample buffer, fractionated in 12.5% or 15% gels by SDS–PAGE and electro‐transferred to nitrocellulose membrane. Membranes were stained with Ponceau red and immunoblotted with the indicated primary antibodies for: CD9 (1/5000), CD63 (1/5000), CD81 (1/5000) and ADAM10 (1/1000) antibodies provided by E. Rubinstein (Charrin et al., 2001) (Université Paris‐Sud UMRS_935, Villejuif); syntenin (Homemade (Kashyap et al., 2015); 1/3000), Tubulin (Sigma‐Aldrich; 1/10,000), ALIX (Homemade (Baietti et al., 2012); 1/500), HSP70 (Santa Cruz Biotechnology; 1/500), Flotillin‐1 (BD Biosciences; 1/1000), EGFR (Cell Signaling; 1/1000), EpCAM (Santa Cruz Biotechnology; 1/200), Fibronectin (BD Biosciences; 1/5000), Src (Cell signaling; 1/1000), TSP1 (Lab Vision; 1/100), SDC1 intracellular domain (Homemade (Baietti et al., 2012); 1/1000), SDC4 intracellular domain (Abnova; 1/2000), and HRP‐conjugated secondary antibodies (Mouse or Rabbit, Thermofisher scientific; 1/10,000). Signals were visualized using Amersham ECL Prime Western Blotting Detection Reagent (GE Healthcare).

### 3.18 Nanoparticle tracking analysis (NTA)

Concentrations and size distributions of exosomal fractions (100,000 x *g* pellet) isolated from the same number of cells were measured at a dilution of 1:50 in 500 µl of PBS1 with the Nanosight NS300 instrument (Malvern), which was equipped with a 488 nm laser and a sCMOS camera. Three videos of 60 s each were recorded for each sample at 25°C and ‐used to calculate mean values of particle concentration.

### 3.19 Wound‐healing assay

For migration assays, cells were treated with 1 ng/ml of mitomycin (Sigma M4287) overnight to inhibit cell division. Treated cells were plated in Ibidi culture‐inserts (Ibidi Cat‐80206) for 12 h to reach 90%–95% of confluence with or without compounds (100 µM). A wound was created by removing inserts from the dishes. Medium with or without compounds (100 µM) was refreshed to remove dead cells and the cells were observed under an inverted light microscope (Leica SP2) equipped with a camera. Images were taken by MetaMorph software every 10 min for 24 h. Measurements of cell velocity were calculated using the MetaMorph software.

### 3.20 Soft agar colony formation assay

Agarose was melted in water and mixed with DMEM‐F12 medium pre‐incubated at 37°C to reach a 0.6% or a 0.36 % final concentration. A lower layer of 0.6% agarose was deposited in each well of a 96‐well plate except that the first and last rows and columns of the plate were filled with PBS to avoid drying. Agarose was allowed to cool for 15 min before the upper layer containing different concentrations of cells, a final concentration of 0.36% agarose and compounds (100 µM) was added. Compounds were added only once at the beginning of experiment. Each condition was tested in triplicate. Colonies were kept at 37°C in a 5% CO_2_ incubator and daily observed under microscope. At the end of the experiment, cells were tested for their ATP content, an indicator of their metabolic activity, using the Promega kit G7570. In this assay, ATP is used as a co‐factor for a luciferase reaction. Signals were analysed by measuring luminescence with a LUMIstar luminometer (BMG Labtech).

### 3.21 Mammosphere formation assay

MCF‐7 and MCF‐7 SyntKO cells were treated with increasing concentrations of compounds **C58**, **SyntOFF** (50‐100µM) or DMSO 0.2% as control. After 24 h, cells were trypsinized, re‐suspended in normal growth media and washed three times in PBS to remove serum. Cell concentration was determined using the LUNA­FL Automated Fluorescence Cell Counter (Logos Biosystem) and cells were then seeded at 1000 cells/ml in 6‐well Ultra low Adherence plates (Corning Inc., Corning, NY) in MEBM medium (Lonza) supplemented with 5 µg/ml insulin (Sigma‐Aldrich), 20 ng/ml recombinant epidermal growth factor (Sigma‐Aldrich), 1X of B27 supplement (Invitrogen, Carlsbad, CA, USA), 0.5 µg/ml hydrocortisone (Sigma‐Aldrich), 1X of β‐mercaptoethanol and 1% penicillin/streptomycin (Gibco). Fresh medium was added into the plates every 3–4 days. The number of spheres (diameter > 50 µm) was evaluated under microscope on days 7–10. Sphere formation was calculated as the number of spheres divided by the original number of cells seeded and normalized to control.

## 4 RESULTS

### 4.1 In vitro evaluation of a PDZ‐focused compound library

**FIGURE 1:**
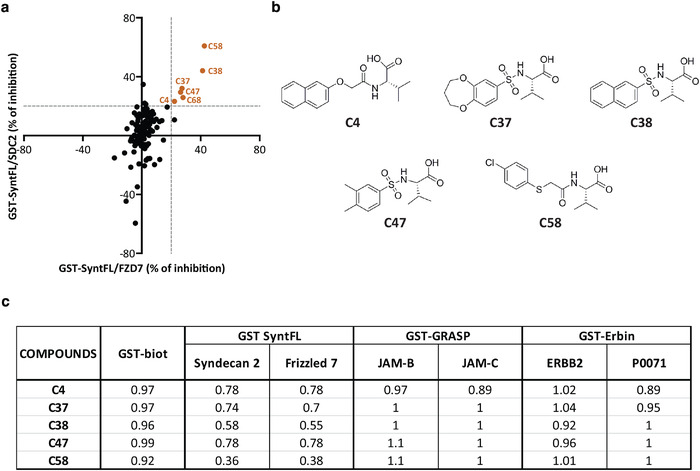
Biochemical assays identify five structurally related active compounds amongst 139 in silico selected potential syntenin PDZ2 inhibitors. (a) Dot plot illustrating the percentage of inhibition of the syntenin‐SDC2 (Y‐axis) and syntenin‐Frizzled 7 (X‐axis) interactions, as determined experimentally by HTRF for the 139 potential inhibitors selected in silico. Compounds that interfere with the FRET signal in a non‐specific manner were previously excluded from the analysis. See table S1, column 'GST‐biot' for more details (b) Chemical structure of the five structurally related compounds (**C4**, **C37**, **C38**, **C47** and **C58**) inhibiting syntenin PDZ interactions. See table S1 for more details. (c) Selectivity of syntenin PDZ inhibitors. Values correspond to the ratio of the signal in the absence of compound (FRET for the interaction) divided by ratio of the signal in the presence of 400µM compound. Compound number (column 1), effect on biotinylated‐GST (column 2), on syntenin‐SDC2 or syntenin‐Frizzled 7 (column 3–4), on GRASP55‐JAM‐B or GRASP55‐JAM‐C (column 5 or 6), and on Erbin‐ERBB2 or Erbin‐P0071 interactions (column 7 or 8). Biotinylated GST (GST‐biot) was used to test for the effects of the compounds on the FRET signal (irrespective of effects on any interaction). Values indicate minor effects (below 10 % difference) validating the approach for each individual compound. HTRF assays on GRASP55 and Erbin PDZ interactions were performed to address the selectivity of the compounds. Note that **C58** selectively affects syntenin PDZ interactions, while compound **C68** also inhibits GRASP‐JAM‐B interaction

**FIGURE 2:**
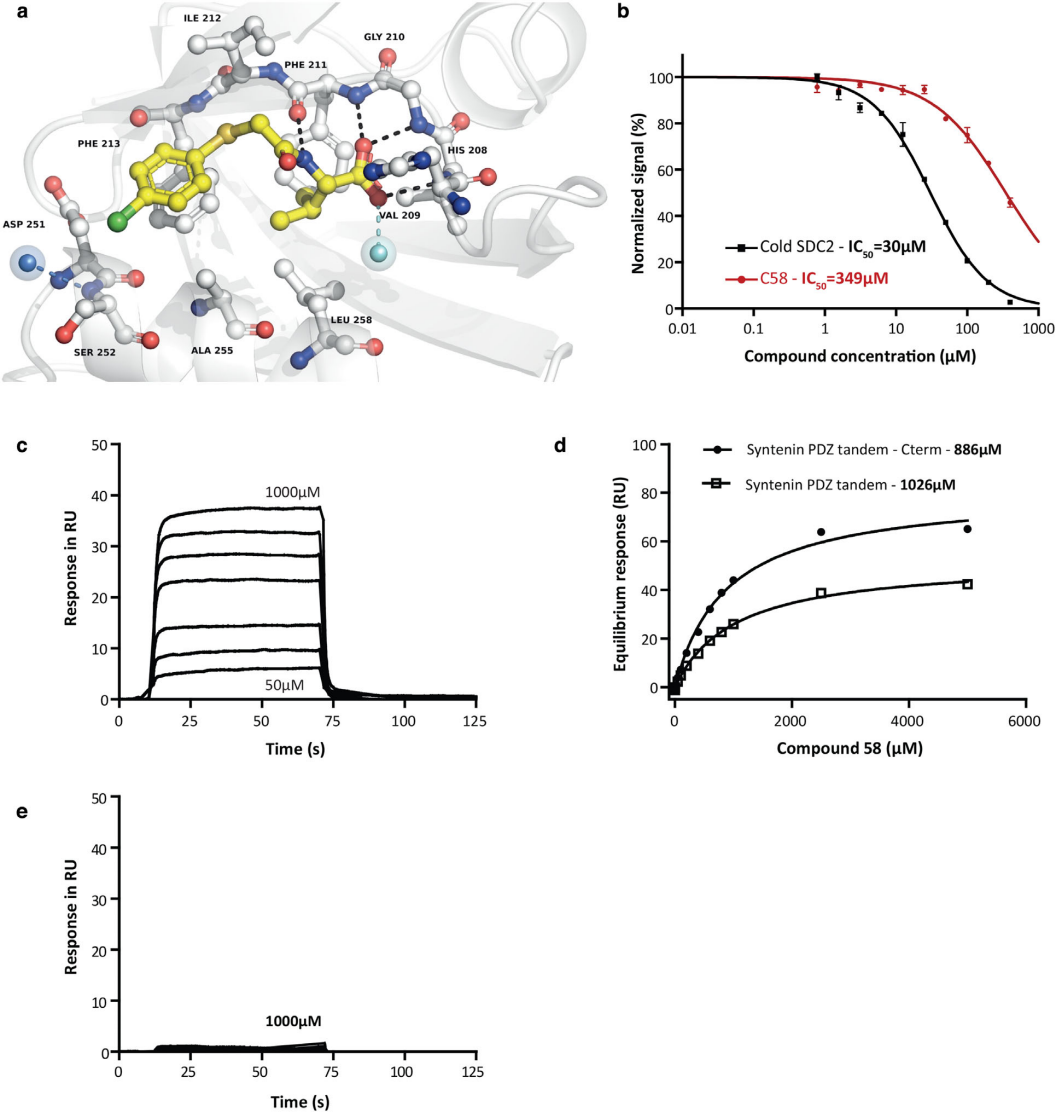
Characterization of C58‐syntenin interaction. (a) Details of the interaction of compound **C58** with the PDZ tandem of syntenin as determined by crystallographic approach (PDBID: 6R9H). Structures are represented as ribbons while compounds are represented as balls and sticks. Hydrogen bonds are represented as black dashes. Water molecules seen in the cavity are shown as blue spheres. One water molecule establishes a direct hydrogen bond with C58 and is highlighted as a cyan sphere. (b) Determination, by HTRF assays, of the half maximal inhibitory concentration (IC_50_) of the SDC2 cognate peptide (black) and compound **C58** (red) for syntenin‐SDC2 complexes. (c) BIAcore sensorgrams (subtracted for GST reference channel) illustrating the binding of increasing concentrations (as indicated) of compound **C58** (50µM ‐1000 µM) to N‐terminally biotinylated‐syntenin PDZ tandem immobilized on a streptavidin sensorchip (d) Langmuir graph showing the binding (in resonance units, RU) of compound **C58**, at equilibrium as observed in BIAcore (compound **C58** was in PBS), to biotinylated‐syntenin PDZ tandem (open squares) or biotynlated‐syntenin PDZ tandem plus c‐terminal domain (plain dots), as a function of compound **C58** concentration. (e) BIAcore sensorgrams (subtracted for GST reference channel) illustrating the absence of binding of compound **C58** (50µM ‐1000 µM) to biotinylated GST‐GRASP PDZ tandem

The search strategy for PDZ2 syntenin inhibitors was based on the PDZ targeted library developed from Enamine compound collection. The PDZ targeted library was developed to cover virtually all PDZ domains. Consequently, to create the syntenin PDZ2 focused library we used two independent paths: docking of the PDZ targeted library against structures of syntenin PDZ2 available in Protein Data Bank and a random selection from the PDZ targeted library. The docking has produced 21 virtual hits, which were added to 118 randomly selected compounds (10% of the pan PDZ library). The resultant set of 139 compounds, syntenin PDZ library was subjected to experimental validation. The primary screen was performed at 400µM using a previously validated Homogeneous Time Resolved Fluorescence (HTRF) assay (Milhas et al., 2016). HTRF is based on Fluorescence Resonance Energy Transfer (FRET) between two fluorescent reagents. In the present case, Anti‐GST‐Terbium antibody and streptavidin‐bound Dynamic2 were used as donor and acceptor respectively. Interactions between GST PDZ‐fusion proteins and biotinylated peptides containing PDZ binding motifs were challenged with the potential inhibitors. In parallel, control experiments were performed with a biotinylated‐GST to verify that compounds were not interfering with the FRET signal in a non‐specific manner. To identify inhibitors of syntenin PDZ interactions, we tested for the effect of the 139 compounds on syntenin‐SDC2 and syntenin‐Frizzled7 complexes (Figure 1a; Table S1). When the HTRF signal dropped below 0.9, inhibition was considered as effective. Five structurally related compounds (valine–like analogues mimicking the last hydrophobic residue of a PDZ binding motif), namely **C4**, **C37**, **C38**, **C47** and **C58 (**Figure 1b) were identified to inhibit syntenin PDZ interactions while having no significant effect on control (biotinylated‐GST) FRET signals. Noteworthy, these compounds had no effect on other PDZ complexes like GRASP55‐JAM‐B or GRASP55‐JAM‐C and Erbin‐Erbb2 or Erbin‐P0071 (Figure 1c). Interestingly, compounds **C38** and **C58** specifically inhibit Syntenin‐PDZ complexes, respectively, to an extent of 40% and 60% (Figure 1c). To validate and characterize the binding mode of the most potent compound **C58** with its target protein, syntenin crystals were soaked with the compound. X‐ray crystal structure of **C58** bound to syntenin was resolved (PDB code 6R9H) and revealed that **C58** is located within the syntenin‐PDZ2 domain (Figure 2a). The phenyl ring of fragment **C58** shows a strong face‐to‐face π‐stacking with Phe213, its nitrogen atom making a hydrogen‐bonding interaction with the backbone carbonyl of Phe211 and its carboxylic acid making the expected canonical hydrogen‐bonding interactions with the backbone nitrogen of Val209, Gly210 and Phe211. **C58** was further characterized in dose‐response HTRF assays testing syntenin‐SDC2 interaction and the IC_50_ was determined to be 350 µM (Figure 2b).

The binding of **C58** to syntenin was also validated in surface plasmon resonance experiments (BIAcore) and in this experimental setting apparent K_D_ values were determined to be 886 µM and 1026 µM for syntenin‐PDZ tandem plus C‐terminal domain and syntenin‐PDZ tandem (without C‐terminal domain) respectively (Figures 2c & 2d). Consistent with the selectivity of **C58** for syntenin, no binding could be observed in BIAcore between this compound and GRASP55‐PDZ tandem (Figure 2e).

### 4.2 In vitro validation of syntenin C58 inhibitor

**FIGURE 3:**
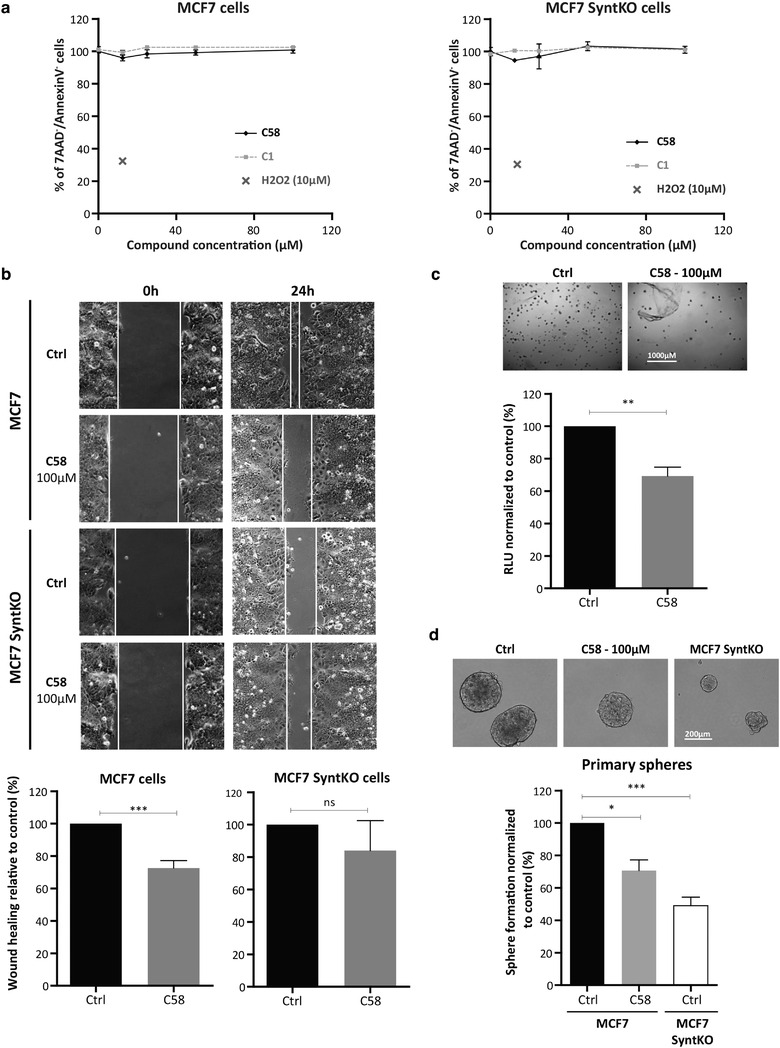
Compound C58 impairs MCF7 cell proliferation, migration and primary sphere formation, pending on syntenin expression. (a) Wild‐type MCF‐7 cells (MCF‐7 cells) and MCF‐7 cells with syntenin knock‐out (CRISPR‐Cas9 approach / MCF‐7 SyntKO cells) were treated with increasing concentration (0.1‐100µM) of compounds **C58** or **C1** (taken as negative control) for 48h. Cell viability was assessed by flow cytometry. (b) (Upper part) Wide‐field micrographs illustrating wound healing assays performed on MCF‐7 and MCF‐7 SyntKO cells treated with vehicle (PBS) or compound **C58** (100µM). We monitored the ability of cells to migrate into the scratch by photographing the same area at the beginning of the experiment (0h left) or 24 h later (right) with an inverted microscope equipped with a digital camera. (Lower part). Wound closure was expressed relative to control (vehicle treated cells), bars represent mean value ± SD of three independent experiments. Statistical analysis was performed using Student's *t*‐test (***P < 0.001). C. (Upper part) Representative micrographs showing MCF‐7 cells colony formation in soft agar comparing samples pretreated with vehicle (PBS) or compound **C58** (100µM). The number of colonies was evaluated after 7 days. (Lower part) The bar graph indicates the percentage of luciferase activity relative to control (vehicle). Values correspond to mean ± sd of three independent experiments. Statistical analysis was performed using Student's *t*‐test (**P < 0.01). (d) MCF7 and MCF7 SyntKO cells were treated with vehicle (DMSO 0.2%) or with compound **C58** (50 & 100µM) and seeded at the same density to form mammospheres. Micrographs are representative of the mammospheres formed after after seven days in culture (upper part). The number of spheres (diameter > 50 µm) was evaluated under a microscope on days 7–10. Three independent experiments were performed. Sphere formation was calculated as the number of spheres divided by the original number of cells seeded and normalized to control ± sd. Statistical analysis was performed using the one‐way analysis of variance (ANOVA) with a Bonferroni posttest (*P < 0.05; **P < 0.01; ***P < 0.001) (lower part)

In wild‐type MCF‐7 breast carcinoma cells and in MCF‐7 cells with (CRISPR/Cas9‐engineered) syntenin‐KO (SyntKO), compound **C58** is not toxic at concentrations up to 100µM (Figure 3a). While such concentration of **C58** decreases MCF‐7 cell migration, no such effect was observed in MCF‐7 cells that are KO for syntenin (MCF‐7 SyntKO) (Figure 3b), documenting the biological specificity of **C58** effects. **C58** also decreases colony formation (Figure 3c) and mammosphere formation (Figure 3d) in wild‐type MCF‐7 cells. Interestingly, syntenin‐KO in MCF‐7 cells mimics the effects of **C58** treatment (Figure 3b micrographs, d). Taken together, our data indicate that syntenin function can be selectively and specifically inhibited by a small compound targeting its PDZ2 domain. Yet, SDC2 cognate peptide outperforms our best chemical compound by a factor 10 in HTRF (Figure 2b) indicating room for improvement.

### 4.3 Hit optimization increases the affinity for syntenin‐PDZ2 domain

In an attempt to optimize **C58** affinity for syntenin, we synthesized 16 analogues covering three different structural modifications (thioether vs. ether, chlorine vs. hydrogen and valine vs. alanine moieties) and tested their ability to impair the formation of syntenin‐SDC2 complex, using HTRF assays. Yet, this approach failed to identify any better compound. Surprisingly, a 'thioether to ether' analogue proved to be almost inactive, highlighting the difficulty to favourably interact with the protein backbone using our chemical core. An alternative strategy was therefore considered, aimed at both optimizing strain energy of the fragment and decreasing desolvation penalty upon binding from the protein side. Molecular modelling studies, relying on the X‐ray structure of **C58 (**Figure 2a) and MOE/Pymol tools, suggested that addition of one carbon spacer in the compound structure could maintain both the canonical binding mode and, the face‐to‐face π‐stacking with Phe213, while slightly increasing the distance between the hydrophobic thioether‐chlorophenyl moiety and the polar protein backbone. Moreover, better torsion angles, closer to optimal geometry (60°), with favourable staggered conformation on the flexible carbohydrate moiety, were also anticipated for this **C58** analogue.

**FIGURE 4:**
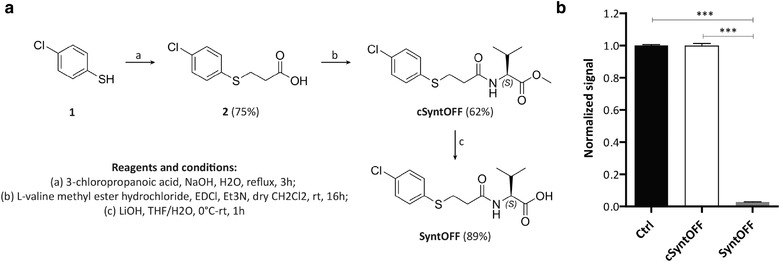
SyntOFF, a potent C58 derivative. (a) Chemical synthesis scheme of the compound **SyntOFF** and its intermediary **cSyntOFF**. Yields are indicated in percentage (b) Activities of 400 µM **SyntOFF** and **cSyntOFF** (also further used as negative control) as determined in HTRF assay testing for inhibition of SDC2‐syntenin interaction. Values correspond to mean ± sd of three independent experiments. Statistical analysis was performed using the one‐way analysis of variance (ANOVA) with a Bonferroni posttest (***P < 0.001)

**FIGURE 5:**
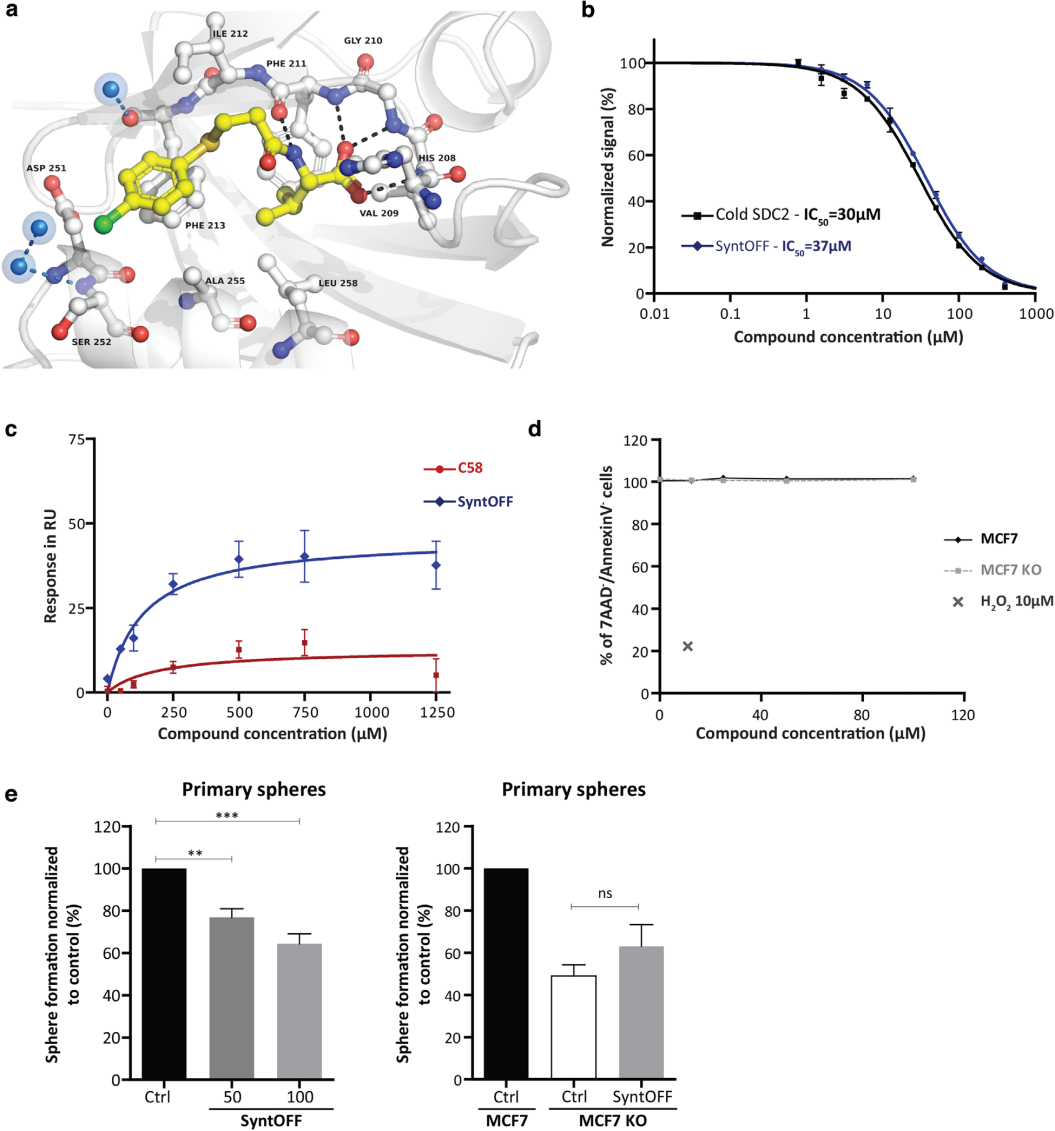
Characterization of SyntOFF biological activity and interaction with syntenin. (a) Details of the interaction of compound **SyntOFF** with the PDZ tandem of syntenin as determined by crystallographic approach (PDBID: 6RLC). Structures are represented as ribbons while compounds are represented as ball and stick. Hydrogen bonds are represented as black dashes. Water molecules seen in and around the cavity are shown as blue spheres. (b) Determination, by HTRF assays, of the half maximal inhibitory concentration (IC_50_) of the SDC2 cognate peptide (black) and SyntOFF (blue) for syntenin‐SDC2 complexes (c) Plot comparing the binding of compound **C58** and **SyntOFF** to biotinylated‐syntenin PDZ tandem plus C‐terminal domain, as a function of compound concentration. Values correspond to binding at equilibrium as observed in BIAcore (compounds were in DMSO). (d) MCF7 and MCF7 SyntKO cells were treated with increasing concentration (0.1‐100 µM) of **SyntOFF** for 48h. Cell viability was assessed by flow cytometry. 10 µM H_2_O_2_ treatment was used as positive control. (e) MCF7 cells (left panel) and MCF7 or MCF7 SyntKO (right panel) cells were treated with vehicle (Ctrl, DMSO 0.2%) or with **SyntOFF** (50 & 100 µM, 100 µM right panel) and seeded at the same density to form mammospheres. The number of spheres (diameter > 50 µm) was evaluated under a microscope on days 7–10. Three independent experiments were performed. Sphere formation was calculated as the number of spheres divided by the original number of cells seeded and normalized to control ± sd. Statistical analysis was performed using the one‐way analysis of variance (ANOVA) with a Bonferroni posttest (*P < 0.05; **P < 0.01; ***P < 0.001)

A new compound with one carbon spacer, further called **SyntOFF**, was synthesized in three steps from the commercial four‐chlorothiophenol (Figure 4a, Figure S1). A first conversion to 3‐(4‐chlorophenyl thio)propanoic acid by S‐alkylation gave derivative **2**, which was condensed with the L‐valine methyl ester hydrochloride leading the methyl ester intermediate **cSyntOFF**. Then, **cSyntOFF** was hydrolysed to the desired compound **SyntOFF** with an excellent yield. **SyntOFF** accordingly inhibited the syntenin‐SDC2 interaction by 96% at 400 µM in HTRF, while the methyl ester intermediate and very close analogue **cSyntOFF** proved to be inactive (Figure 4a & 4b, Figure S1). The X‐ray crystal structure of compound **SyntOFF** in complex with syntenin was also resolved (PDB code 6RLC) indicating, as anticipated, a canonical binding mode with the carboxylate binding loop (Val109/Gly210/Phe211) into syntenin‐PDZ2 domain (Figure 5a). The small structural modification, consisting in the introduction of a single methylene group as a spacer, resulted in a > 10‐fold improvement of the affinity as compared to **C58** (IC_50 =_ 37 µM). **SyntOFF** reached the reference ligand efficiency (Hopkins, Groom, & Alex, 2004) threshold of 0.30 kcal/mol/atom, starting from the **C58** moderate 0.24 kcal/mol/atom value, highlighting the optimal introduction of an additional one carbon spacer in the structure of the **C58** (compare Figure 5b and 2b). **SyntOFF** appeared selective for syntenin, not affecting Erbin‐P0071 (Figure S2a) or GRASP55‐JAM‐B complexes in HTRF (Figure S2b). In BIAcore, using DMSO as vehicle, **SyntOFF** displayed a K_D_ value of 130 µM for syntenin‐PDZ tandem plus C‐terminal domain (Figure 5c). In cellular assays, **SyntOFF**, like **C58**, is non‐toxic up to 100 µM (Figure 5d). Stability properties were evaluated (Table S2), it appears that **SyntOFF** is stable in PBS pH7.4 (solubility > 7.3 mM) and also in murine plasma (81% of remaining compound after 2h). Moreover, **SyntOFF** decreases MCF‐7 mammosphere formation more efficiently than does **C58** (Figure 5e, left to compare to Figure 3d). Of note, **SyntOFF** had no effect when MCF‐7 cells were KO for syntenin expression (Figure 5e, right).

### 4.4 Syntenin‐PDZ2 domain inhibitor regulates exosome cargo

Next, we investigated the effect of **C58**, **SyntOFF** and the inactive control compound **cSyntOFF** (Figure 4a) on the production and the composition of exosomes. As it was previously shown that the PLD2 inhibitor (CAY10594; 10 µM) affects the secretion of exosomal markers including syntenin (Ghossoub et al., 2014), we included this enzymatic inhibitor as control, in parallel to our syntenin‐PDZ inhibitors. Nanoparticle tracking analyses (NTAs) revealed that none of the present syntenin‐PDZ2 inhibitors (100 µM) affects the size or the number of secreted nanoparticles, whereas PLD2 inhibitor clearly decreases their number (Figure S3a and S3b).

**FIGURE 6:**
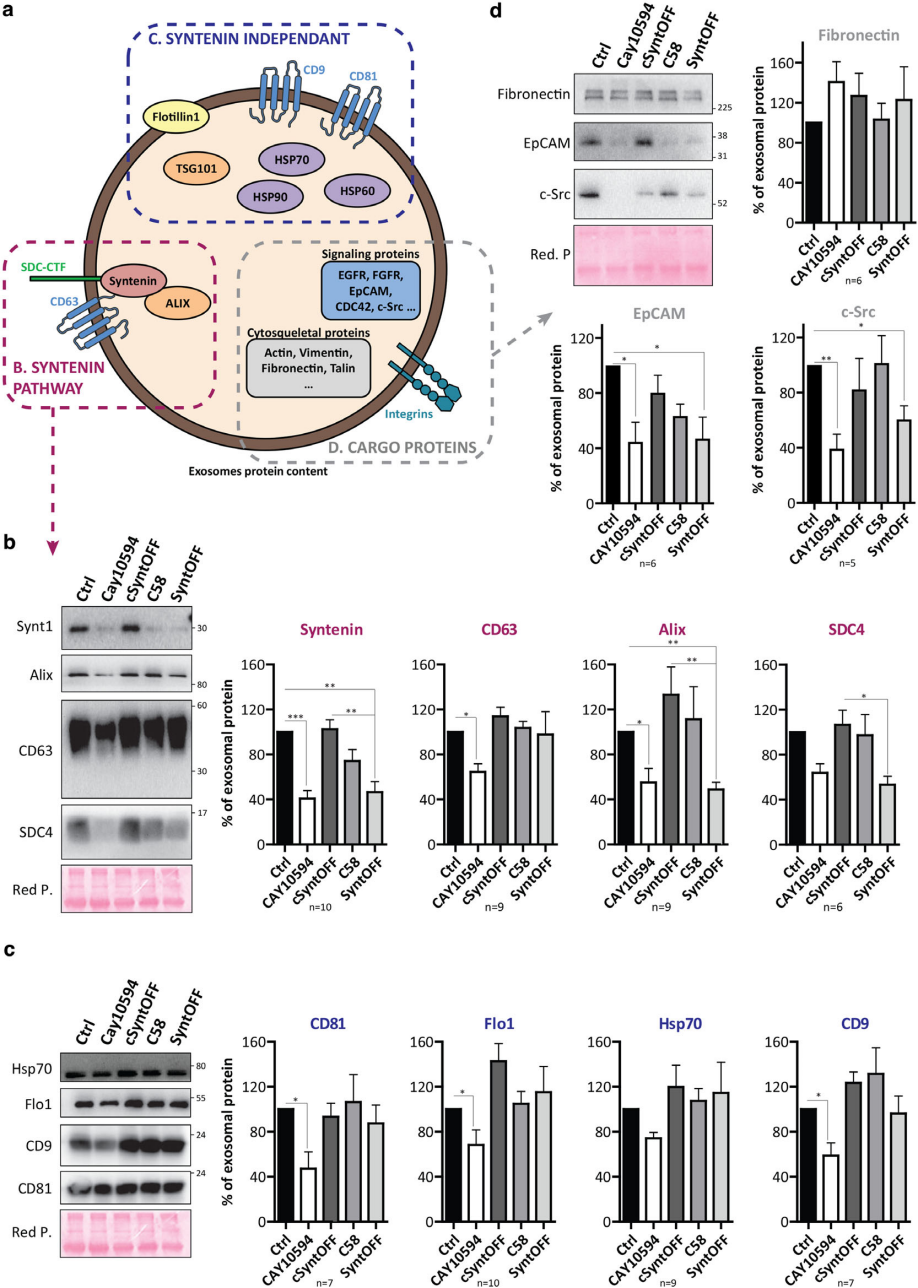
SyntOFF regulates the loading of exosomes with syntenin‐dependent cargo and EpCAM. (a) Schematic representation of an exosome highlighting which markers were previously shown to rely on syntenin (red, syntenin pathway) or to be unaffected by syntenin (blue, syntenin independent) for their secretion in MCF7 exosomes. The scheme also represents additional exosome cargo (cargo proteins, among which Fibronectin, EGFR and EpCAM investigated in this study). (b‐d) MCF7 cells were treated with DMSO vehicle (Ctrl) or the indicated compound (CAY10594 10 µM; **cSynOFF**, **C58**, or **SyntOFF** at 100 µM) for 16 h before analysis. Total cell lysates (See supplementary figure S4) and the corresponding exosomes were analyzed by western blot, tracing different markers, as indicated. Histograms represent mean signal intensities (±SEM) in exosomes, relative to controls (Ctrl). Data were obtained from n independent experiments, as indicated. Statistical analysis was performed using the one‐way analysis of variance (ANOVA) with a Bonferroni posttest (*P < 0.05; **P < 0.01; ***P < 0.001)

Exosomal fractions released by cells treated with syntenin‐PDZ2 or PLD2 inhibitors were then analysed by western blot, investigating the effect of the different compounds on both syntenin‐dependent and ‐independent exosomal markers (Figure 6a). The amounts of exosomal syntenin, ALIX and SDC4 C‐terminal fragment (CTF) were significantly decreased when cells were treated with 10 µM PLD2 inhibitor or 100 µM **SyntOFF**, but not with 100 µM C58 or the control compound **cSyntOFF** (Figure 6b). PLD2 inhibitor also markedly decreased exosomal markers like CD9, CD81 and Flotillin‐1, but that was not the case for **SyntOFF** (Figure 6c). Noteworthy, cellular levels remained unaffected, regardless of the treatment (Figure S4a and S4b). Finally, we also examined whether syntenin inhibitors affect the loading of exosomes with signalling or adhesion proteins (Figure 6a). Clearly, both **SyntOFF** and PLD2 inhibitor markedly decrease the incorporation of EpCAM and c‐Src into exosomes (Figure 6d), but leave fibronectin (Figure 6d), EGFR, ADAM10 or TSP1 (Figure S5a) signals unaffected. Here again, drugs did not impact on the cellular levels (Figure S4c and S5b). Taken together, our data indicate that inhibition of syntenin PDZ2 domain, while having no major effect on exosome number or cellular protein levels, can impact on the secretion of specific exosome cargo. They also highlight that EpCAM loading into exosomes depends on syntenin.

## 5 DISCUSSION

In this study, we have developed a novel syntenin inhibitor, **SyntOFF**, which specifically binds syntenin, and serves as an antagonist of syntenin‐PDZ2 domain, interfering with cell migration, proliferation and mammosphere formation, as well as inhibiting the loading of exosomes with specific cargo.

By screening 139 in silico‐selected compounds, we first identified a family of five structurally related compounds as potential inhibitors of syntenin PDZ interactions. All compounds similarly affected the interaction of syntenin with Frizzled 7 and SDC2 (Figure 1), two well‐documented interactors of syntenin PDZ2 domain (Egea‐Jimenez et al., 2016; Grembecka et al., 2006). As shown in Figure 1, these five inhibitor candidates share, at one end, a common pharmacophore (a valine‐like residue) mimicking the last hydrophobic residue at the C‐terminus of cognate peptides. They also present, on another end, a variously substituted aromatic core. The **C58** inhibitor was selected for further investigations because it yielded the highest extinction of the HTRF signal. In complementary biophysical experiments, **C58** proved to selectively bind to syntenin (Figure 2b‐c) and not to GRASP55 PDZ domains (Figure 2e). Moreover, co‐crystallization experiments indicated that **C58** binds syntenin PDZ2 but not PDZ1 domain (Figure 2a). Noteworthy, when used at a concentration of 100 µM (three times below the IC_50_ calculated by HTRF), **C58** exerts biological effects on cells expressing syntenin, but not on cells where syntenin was depleted by CRISPR‐Cas9 approach (Figure 3). These data suggest that small compounds (or drug fragments) can achieve biological specificity. Interestingly, **C58** had no toxic effects at working concentrations (Figure 3a). Because **C58** displays an IC_50_ one order of magnitude higher than the cognate peptide SDC2 (Figure 2b), we surmised there must be room for improvement. Yet, classical chemical approaches, relying on the growing concept from Fragment Based Drug Discovery completely failed. However, an optimization strategy, based on crystallographic data, enabled the development of **SyntOFF** where the introduction of an additional methylene group (Figure 4) improved the affinity 10‐fold compared to **C58** (**compare** Figure 2b & 5b). As expected, **SyntOFF** also interacts with syntenin PDZ2 and not PDZ1 domain according to crystallography data (PDB 6RLC and Figure 5a). Consistent with the gain in affinity, **SyntOFF** appeared more effective than **C58** in cellular cancer assays (Figure 5e). **SyntOFF** was also non‐toxic at concentrations where it was biologically active (Figure 5d). As observed for **C58**, **SyntOFF** biological effects were solely significant on cells expressing syntenin (Figure 5e). Of note, PDZ interactions are promiscuous (Ivarsson, 2012) and PDZ binding motifs are known to bind multiple PDZ proteins. Therefore, the selectivity and the biological specificity observed for **C58** and **SyntOFF** were not anticipated. Interestingly, although it is unclear to date which ligands might directly and efficiently interact with syntenin PDZ1 domain (Grembecka et al., 2006), Fisher's group recently developed a syntenin‐PDZ1 domain‐targeted small molecule. This molecule reduces the invasive potential of glioblastoma cells (Kegelman et al., 2017), as well as the invasiveness and the migration of prostate cancer cells (Das et al., 2019). We thus have evidence that syntenin PDZ interactions can be targeted by small organic chemicals, be it with modest affinity in the same range that the cognate peptides. Besides, such chemicals compounds are non‐toxic at concentrations where they display biological effects and specificity.

When used at 100 µM, **SyntOFF**, but not **C58** or the negative control compound **cSyntOFF** (unable to make the canonical H‐bonds network with the carboxylate binding loop into syntenin PDZ2 domain) reduced the amounts of exosomal syntenin, ALIX and SDC4 CTF (Figure 6b). Moreover, **SyntOFF** does not seem to affect other exosome populations (Figure 6c). These results confirm the success of the early stages of our optimization and illustrate that **SyntOFF** significantly alters the syntenin‐dependent exosomal pathway. Yet, to meet current standards in drug discovery, we need to improve affinity by one order of magnitude. For these reasons, we will continue to work on syntenin PDZ2 chemical inhibition and optimization of compounds to improve their molecular and pharmacological properties by performing a modelling‐assisted fragment growing process.

**SyntOFF** specifically targets and antagonizes the Syntenin‐PDZ2 domain in vitro, which might explain why our compound suffices for effectively and specifically inhibiting syntenin‐SDC, including exosomal pathways. Interestingly, we noticed that pharmacological inhibition of the syntenin‐PDZ2‐domain does not affect the number of the nanoparticles secreted, whereas our group had previously shown that syntenin controls the number of exosomes made/released. Indeed, using syntenin RNAi‐treated MCF‐7 cells, Baietti et al. had observed that loss of syntenin leads to a ∼50% reduction in the number of particles with a diameter of 30–110 nm. This effect was further validated by confocal fluorescence spectroscopy assessing the effects of syntenin overexpression on the detection of mGFP–CD63 in exosomes, with a two to three‐fold increase in the number of the fluorescent peaks. These results suggest that the syntenin‐PDZ2 inhibitor affects syntenin‐exosomal loading without altering the endosomal membrane budding. The sequestration of cargo in specific endosomal membrane domains and the budding of these membranes to create intraluminal vesicles and multi‐vesicular endosomes are processes that mechanistically are closely intertwined. Yet the finding with the inhibitor is consistent with the notion that the stoichiometry between membrane unit area, membrane cargo and budding machinery is not constant, the PDZ2 inhibitor leading to the production of relatively ‘cargo‐depleted’ intraluminal vesicles, and from there ‘cargo‐depleted’ exosomes. That notion (of vesicles of heterogeneous compositions) is commonly accepted in the fields of virology, studying the assembly of viruses and defective, virus‐like particles. Likely, more potent PDZ2 inhibitors, more effective at thinning and disrupting syndecan‐syntenin lattices on endosomal membranes, will ultimately prove to also affect exosome numbers. Alternatively, exosome uptake mechanisms and net exosome accumulations in peri‐cellular spaces might also be exosome‐ and cargo‐specific, and differentially depend on syntenin functions in recipient cells. Further investigations are needed to clarify these possibilities.

Consistent with our previous work, we noticed that pharmacological inhibition of the syntenin‐PDZ2‐domain markedly decreases the incorporation of c‐Src into the exosomes (Imjeti et al., PNAS 2017). Finally, our results strongly suggest that EpCAM is sorted to exosomes via the syntenin machinery (Figure 6d), depending on determinants the path has in common with syntenin‐mediated SDC sorting. However, EpCAM finding must be confirmed, also using complementary approaches, and it remains to be established how this sorting proceeds: that is, whether direct interactions with syntenin are involved (i.e. via PDZ2, the PDZ‐BM of EpCAM composing alternative bait for syntenin), and whether, like that of CD63 (Baietti et al., 2012), EpCAM sorting to exosomes might depend on the assistance of SDC (CD63 binding primarily via PDZ1, SDC via PDZ2). EpCAM was originally identified as a marker for carcinoma and its role includes diverse processes such as signalling, cell migration, proliferation, and differentiation (Keller et al., 2019). Some studies suggest that the levels of exosomal EpCAM could constitute a biomarker of ovarian and pancreatic cancers (Im et al., 2014) (Castillo et al., 2018). Proteomic analysis of EVs isolated from colon carcinoma cell‐derived organoids even revealed a distinct population of exosomes according to EpCAM expression and showed a colocalization of EpCAM with CD44 and claudin 7, proteins that are known to promote tumour progression (Tauro et al., 2013). In accordance with previous data from our laboratory concerning syntenin‐dependent cargo (SRC, FGF, Frizzled 7), this result reinforces the notion of the syntenin‐SDC exosomal pathway as an important player in cancer progression.

Overall, we show that Syntenin‐PDZ2 is a promising therapeutic target for inhibiting oncogenic processes and mostly tumour exosomal communication. This study confirmed that syntenin is strongly involved in the loading of exosomes with cargo. Given the versatility of the SDC co‐receptor functions, this potentially pertains to a plethora of pro‐oncogenic proteins that support tumorigenesis and metastatic dissemination. Likely, also further investigations about pro‐tumour miRNA possibly connected with syntenin and its cargo will appear essential for a better understanding of the role of cancer exosomal communication.

## CONFLICTS OF INTTEREST

The authors declare no competing interests.

## AUTHOR CONTRIBUTIONS

All authors provided support for the writing. R. Leblanc carried out HTRF and cell biological work, and wrote the manuscript; R. Kashyap performed HTRF and cell biology experiments; K. Barral contributed to structure–activity relationship studies, crystallization experiments, X‐ray complex structures resolution, and organic syntheses; D. Kovalskyy designed the PDZ focused library; A.L. Egea‐Jimenez did the SPR experiments and protein production for crystallization studies; M.Feracci contributed to crystallization studies and associated calculations; M. Garcia performed chemistry experiments and associated analyses; C. Derviaux contributed to automated HTRF experiments; S. Betzi contributed to protein purification and crystallization studies; R. Ghossoub contributed to cell biology experiments; M. Platonov designed the PDZ focused library; P. Roche helped with the in silico analyses; X. Morelli supervised and conceived the project; L. Hoffer analysed the structural data and carried out the computer‐aided design of organic compounds during the optimization stage; Pascale Zimmermann supervised and conceived the project, and wrote the article.

## Supporting information



Supplementary information

Supplementary information

Supplementary information
