# Supplementary figures and images for: Pharmacological inhibition of syntenin PDZ2 domain impairs breast cancer cell activities and exosome loading with syndecan and EpCAM cargo
Source: J Extracell Vesicles. 2020 Dec 15;10(2):e12039. doi: 10.1002/jev2.12039 (PMC7737769; doi:10.1002/jev2.12039)

A

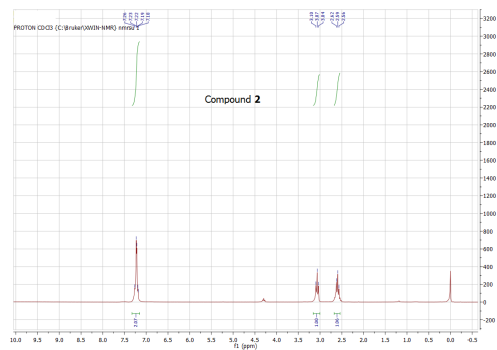

B

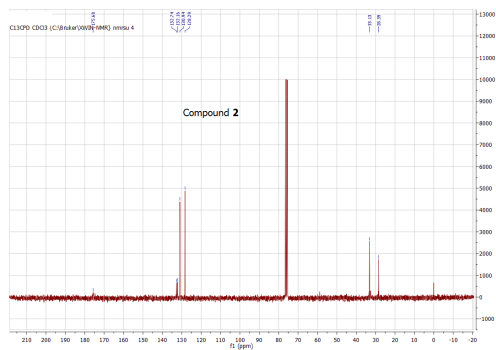

C

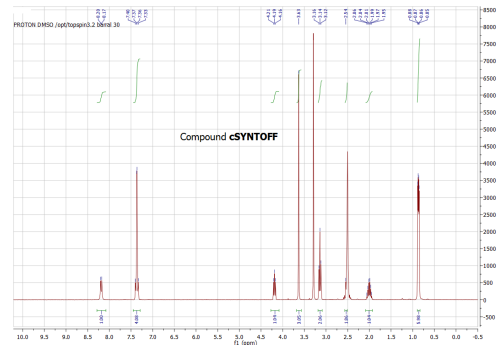

D

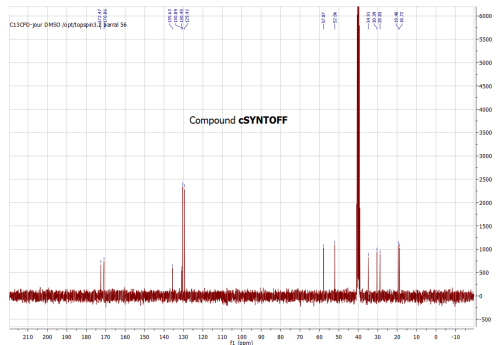

E

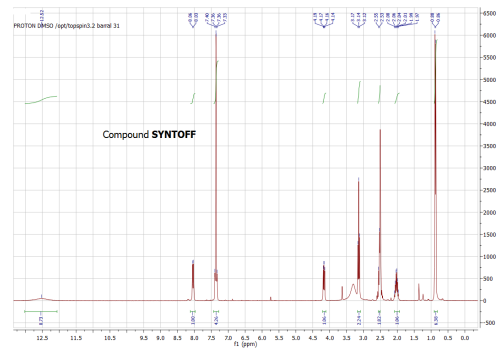

F

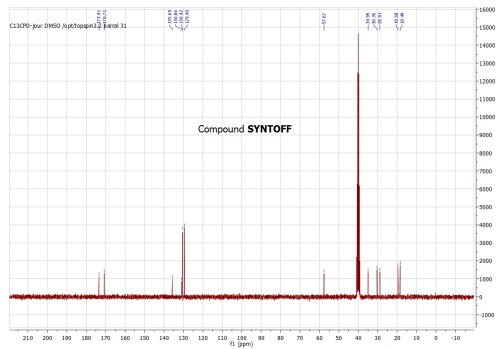

Figure S1

**A**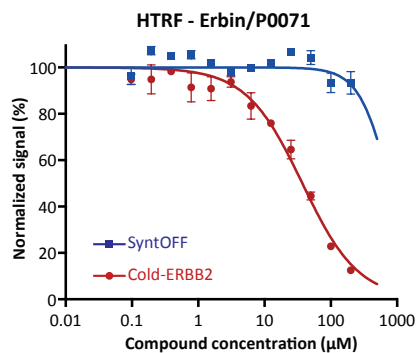**B**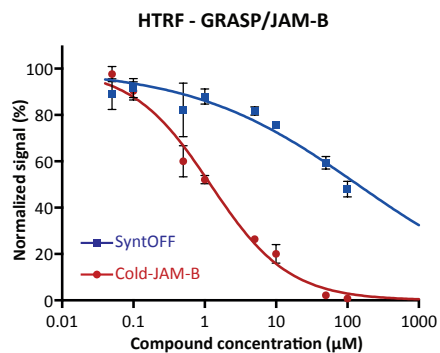**Figure S2**

**A**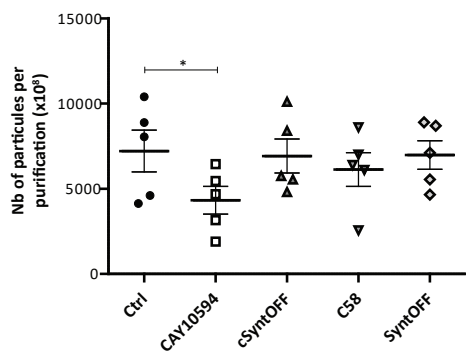**B**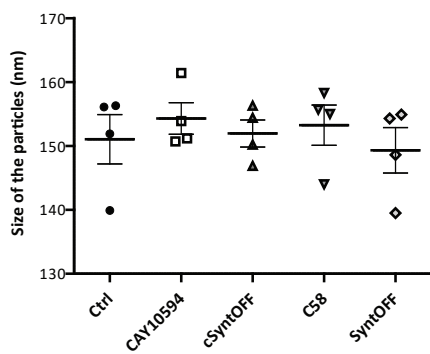**Figure S3**

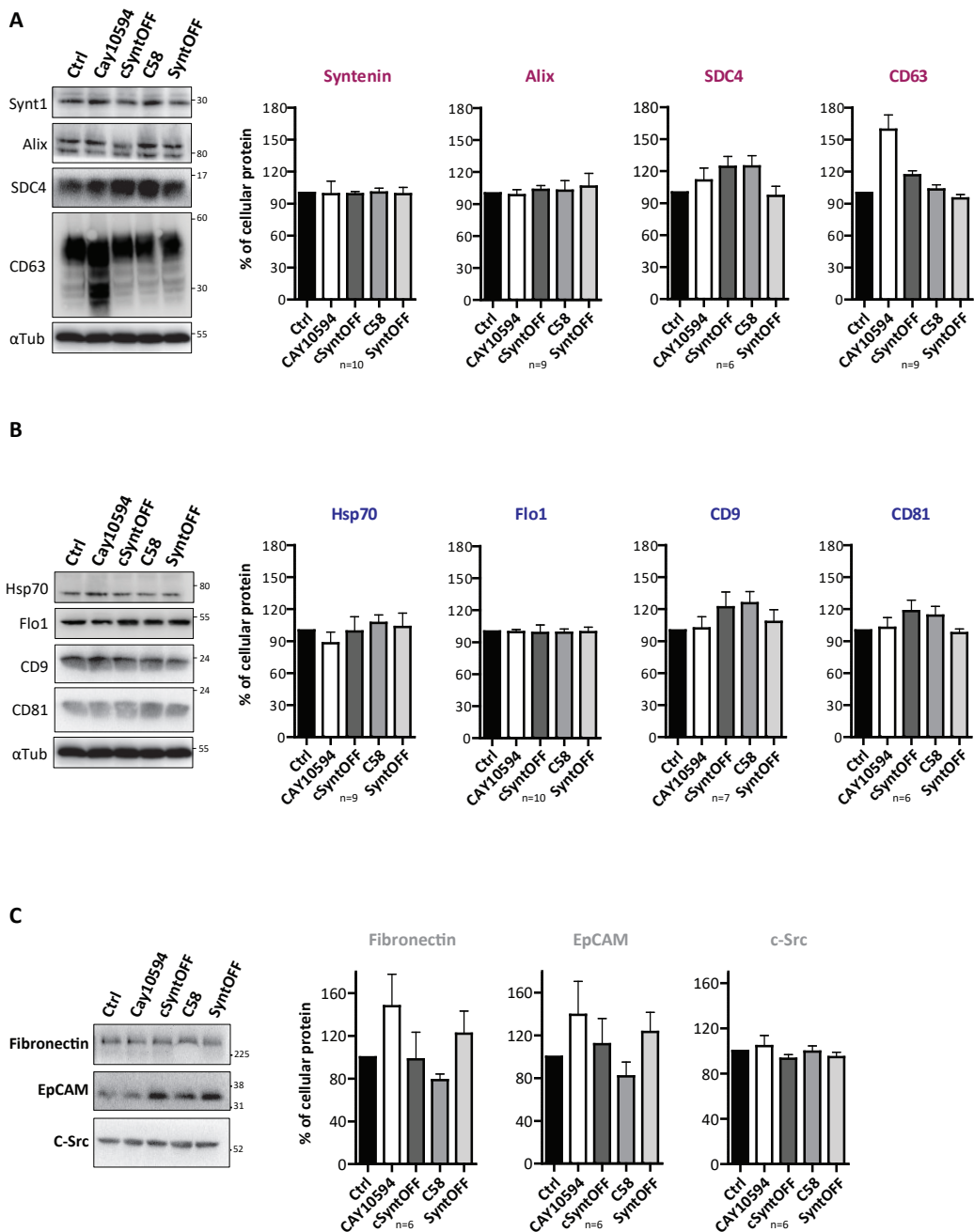

Figure S4

**A**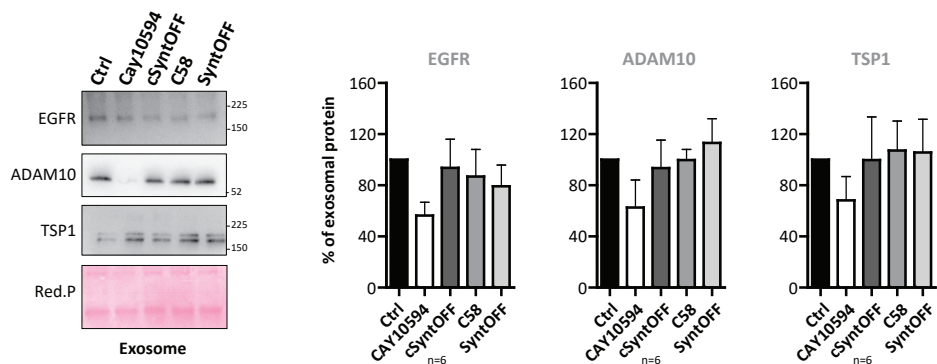**B**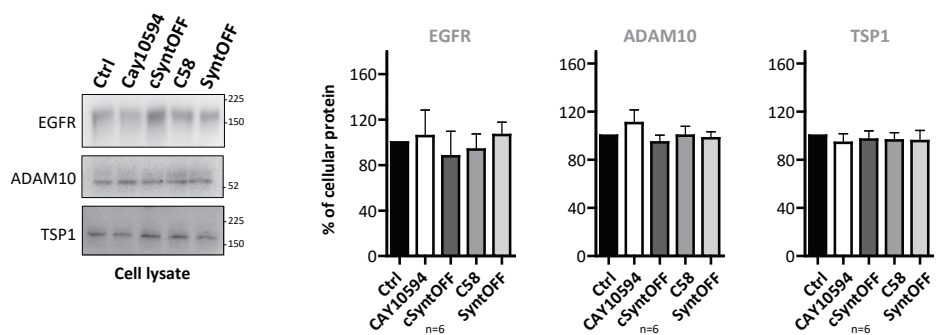**Figure S4**

Supplement: Supplementary file 1 — Supplementary information [file JEV2-10-e12039-s003.pdf]
